# Comparative analysis of the alveolar macrophage proteome in ALI/ARDS patients between the exudative phase and recovery phase

**DOI:** 10.1186/1471-2172-14-25

**Published:** 2013-06-17

**Authors:** Haiyun Dong, Jinxiu Li, Youdi Lv, Yanyan Zhou, Guyi Wang, Shuang Hu, Xiaoyu He, Ping Yang, Zhiguang Zhou, Xudong Xiang, Cong-Yi Wang

**Affiliations:** 1 Intensive Care Unit, Diabetes Center, Second Xiangya Hospital, Central South University, Changsha, Hunan 410011, China; 2 The Center for Biomedical Research, Tongji Hospital, Tongji Medical College, Huazhong University of Science and Technology, 1095 Jiefang Ave., Wuhan, 430030, China; 3 Key Laboratory of Organ Transplantation, Ministry of Education, Ministry of Health, Tongji Hospital, Tongji Medical College, Huazhong University of Science and Technology, 1095 Jiefang Ave, Wuhan 430030, China; 4 Diabetes Center, Second Xiangya Hospital, Central South University, Changsha, China

**Keywords:** ALI/ARDS, Alveolar macrophages, Biomarker, 2D PAGE, MALDI-TOF-MS

## Abstract

**Background:**

Despite decades of extensive studies, the morbidity and mortality for acute lung injury/acute respiratory distress syndrome (ALI/ARDS) remained high. Particularly, biomarkers essential for its early diagnosis and prognosis are lacking.

**Methods:**

Recent studies suggest that alveolar macrophages (AMs) at the exudative phase of ALI/ARDS initiate, amplify and perpetuate inflammatory responses, while they resolve inflammation in the recovery phase to prevent further tissue injury and perpetuated inflammation in the lung. Therefore, proteins relevant to this functional switch could be valuable biomarkers for ALI/ARDS diagnosis and prognosis. We thus conducted comparative analysis of the AM proteome to assess its dynamic proteomic changes during ALI/ARDS progression and recovery.

**Results:**

135 proteins were characterized to be differentially expressed between AMs at the exudative and recovery phase. MALDI-TOF-MS and peptide mass fingerprint (PMF) analysis characterized 27 informative proteins, in which 17 proteins were found with a marked increase at the recovery phase, while the rest of 10 proteins were manifested by the significantly higher levels of expression at the exudative phase.

**Conclusions:**

Given the role of above identified proteins played in the regulation of inflammatory responses, cell skeleton organization, oxidative stress, apoptosis and metabolism, they have the potential to serve as biomarkers for early diagnosis and prognosis in the setting of patients with ALI/ARDS.

## Background

Acute lung injury (ALI) is a common complication resulted from serious infections and traumatic injuries. ALI and its more severe form, the acute respiratory distress syndrome (ARDS), are syndromes manifested by severe hypoxemia, hypercapnia, diffuse infiltration in the lung, and a substantial reduction in pulmonary compliance. Typically, upon the insult of a predisposing factor such as a severe infection, widespread damage of cells and structures of the alveolar capillary membrane can occur within hours to days. Although extensive investigations have revealed the possible underlying molecular mechanisms that offer therapeutic opportunities, the morbidity and mortality for ALI/ARDS, however, remained high. Particularly, biomarkers relevant to ALI/ARDS early diagnosis and therapeutic prognosis are still lacking [1-3].

In the early stage of ALI/ARDS (exudative phase), the patients are usually associated with diffuse alveolar damage, neutrophil infiltration, haemorrhage and the accumulation of a protein-rich pulmonary oedema along with the disruption of the epithelial barrier and injury to the capillary endothelium. On day 5 of ALI/ARDS onset, the patients display typical characteristics for disease recovery (recovery phase) manifested by the variable degree of restoration of the lung function [4]. Given that the alveolar macrophage (AM) functions as a guardian for the alveolar–blood interface against respiratory pathogens, its role in the pathogenesis of ALI/ARDS has recently been highly appreciated [5-7]. It is believed that AM serves as the primary phagocytes responsible for removing the infectious, toxic or allergenic particles from airways [8]. Indeed, once insulted by a severe infection or a traumatic injury, AM synthesizes and secretes a wide array of cytokines such as IL-1β, IL-6, and TNF-α, chemokines, and arachidonic metabolites to initiate, amplify and perpetuate inflammatory responses in the lung, and to recruit activated neutrophils into the alveolar spaces [4]. Interestingly, recent studies have further revealed that AM is also important in resolving inflammation within the airspace [9]. It has been noted that as inflammatory responses to an infectious insult resolve, AM is responsible for the efficient clearance of apoptotic neutrophils by phagocytosis [10], and through which, AM secretes antiinflammatory cytokines such as TGF-β and IL-10 to prevent further tissue injury and perpetuated inflammation [9]. Based on these observations, we herein hypothesize that during the course of ALI/ARDS, AM undergoes a functional property switch manifested by the inflammatory property at the exudative phase (within 24 h of ALI/ARDS onset) and the antiinflammatory property at the recovery phase (day 5 after disease onset), and characterization of biomarkers relevant to this functional switch could be important for ALI/ARDS early diagnosis and prognosis. To test the above hypothesis, we conducted comparative analysis of AM proteome to assess its dynamic proteomic changes during ALI/ARDS progression and recovery. Our studies characterized 27 unique proteins with known functions, and 17 of which were upregulated during the stage of disease recovery, while the rest 10 proteins were highly expressed in the exudative phase. These proteins could be useful for developing biomarkers for early diagnosis and prognosis of patients with ALI/ARDS in the clinical settings.

## Methods

### Patient collection

Fourteen patients with ALI/ARDS (10 males, 4 females, with 18 to 55 years old, average age 48 ± 6.3) were enrolled in the intensive care unit (ICU) at the Second Xiangya Hospital of Central South University from August to December, 2010. All patients were diagnosed according to the following criteria: 1) the presence of ALI/ARDS high risk factors; 2) a sudden onset of rapid respiratory rate and/or respiratory distress; 3) hypoxemia, oxygenation index ≤ 200; 4) inflammatory infiltration of the lung demonstrated by X-ray exam; and 5) clinical exception for cardiogenic pulmonary edema. Those patients with original lung diseases such as obstructive pulmonary disease and severe asthma were excluded from the study. Subjects with recent use of hormone and smokers were also excluded. The above described ALI/ARDS patients were secondary to sepsis due to a severe infection, they met at least two of the following criteria for diagnosis of sepsis, 1) body temperature > 38°C or < 36°C, and 2) heart rate > 90 beats/min, respiratory rate > 20 times/min, PaCO2 < 32 mmHg, WBC count > 12 × 10^9^/L or < 4 × 10^9^/L, or > 10% immature neutrophils. All ALI/ARDS patients admitted to the ICU were in the acute phase (disease onset within 24 h). Informed consent for the research enrollment, fiberoptic bronchoscopy exam and BALF collection was obtained from all selected patients. The study was approved by the Human Assurance Committee at the Central South University.

### Collection of bronchoalveolar lavage fluid (BALF)

BALF from ALI/ARDS patients were obtained on days 1 and 5 upon diagnosis, respectively. Bronchoalveolar lavage (BAL) was carried out according to the guidelines set by the Chinese Society of Respiratory Diseases in 2009. Briefly, the bronchoscope was wedged into the right middle lobe, and through which, 20 ml of sterilized 0.9% saline (100 ml in total for 5 times) were instilled in and then gently aspirated out, and the recovery rate for the stilled saline was more than 40%. The lavage fluid was next filtered through sterilized double-layer of gauze to remove mucus and debris.

### Isolation of alveolar macrophages (AMs)

BALF was centrifuged at 1000 rpm for 10 min at 4°C, and the supernatants were stored at -80°C for further analysis. The cell pellets were washed and resuspended in 10 ml of phosphate-buffered saline (PBS) solution. The number of AMs was estimated employing a grid hemocytometer, while the viability was determined by trypan blue staining. The cells were next mixed with 5 ml RPMI1640 medium supplemented with 10% fetal calf serum in a culture flask, and cultured in a 37°C incubator (5% CO2) for 2 h to allow the cells attach to the bottom. The attached AMs were finally scraped and suspended in PBS solution. After washes, the cells were subjected to preparation of protein lysates.

### Preparation of AM lysates

The above prepared AMs from 14 patients were lysed in Tris–HCl buffer (10 mmol/L Tris–HCl PH7.5, 0.36 ng/mL E-64, 20 mmol/L PMSF, 0.34 mg/mL pepstatin, 5.6 mg/mL benzamidine HCl and 1 mg/ml leupeptin) through 5 rapid freezing-thawing cycles, respectively. DNase I and RNase were next added into each sample and incubated on ice for 10 min, followed by incubation on dry ice for at least another 30 min. The samples were dried using a lypholizer and were finally dissolved in the lysis buffer. Protein concentration for each sample was determined using a protein assay kit (Nanjing Jiancheng Bioengineering Institute, China) using established techniques [11].

### Two-dimensional ployacrylamide gel electrophoresis (2D PAGE)

The AM samples were first separated on pH 3–10 IPG strips according to their isoelectric point by isoelectric focusing as previously reported [12]. Briefly, electrophoresis was conducted at 500 V × 1 h, 1,000 V × 1 h, 3,000 V × 1 h, 6,000 V × 1 h, 8,000 V × 1 h, and up to 70,000 V for a few hours. After isoelectric focusing, the IPG strips were rapidly removed and equilibrated for 15 min in 10 ml of solution A (50 mmol/L Tris–HCl, PH8.8; 6 mol/L Urea; 30% glycerol; 1% SDS; 0.2% DTT and bromophenol blue dye), followed by another 15 min in 10 ml of solution B (50 mmol/L Tris–HCl PH8.8; 6 mol/L Urea; 30% glycerol; 1% SDS; 3% iodoacetamide and bromophenol blue dye). After sequential equilibration in solution with 0.2% DTT and 2.5% iodoacetamide, the strips were transferred to an Ettan DALT vertical electrophoresis system (Amersham Biosciences, Piscataway, NJ USA) as instructed. The gels were run at 30 mA constant current at 15°C until the bromophenol blue dye reached the far edge under the glass plate.

### Two-D gel image analysis and MALDI-TOF-MS

Two-D gel images were analyzed by the Image Master 2DElite 3.01 software. Comparative analysis was conducted to characterize those spots with ≥ 2.5 times expression differences. Only those spots with consistent results in all parallel gels were selected for identity analysis. The selected spots on the gels were next extracted, rinsed in 50% ethyl after destaining, and then dried in a vacuum centrifuge. The samples were next digested with 12.5 mg/L Trypsin and then subjected to matrix-assisted laser desorption/ionization time-of-flight mass spectrometry (MALDI-TOF-MS) analysis for peptide mass fingerprinting (PMF) [12]. Mascot software was used to search the MSDB and NCBlnr protein databases.

### Western blot analysis

AM lysates were prepared using the RIPA lysis buffer with protease inhibitors (Beyotime, China). The loaded proteins (50 μg) were separated by 12% SDS-polyacrylamide gel electrophoresis (SDS-PAGE), and then transferred onto PVDF membranes. After blocking with 5% milk, the membranes were probed with antibodies against S100A9 and HSP27, followed by incubation with a secondary antibody, respectively. The blots were developed using the ECL Plus reagents (Thermo pierce, USA) as previously reported [13]. The intensity of target bands was analyzed by densitometry and normalized by β-actin using the Quantity One software (BioRad, CA, USA).

### Data analysis

Data were summarized as mean and standard deviation. Comparison between groups was carried out using unpaired Student’s *t* test. In all cases, *p* < 0.05 was considered with statistical significance.

## Results

### Clinical features for the selected ALI/ARDS patients

All 14 selected ALI/ARDS patients were actually progressed from severe infections, 6 of which were resulted from severe pancreatitis, 4 patients were progressed from acute suppurative cholangitis, and the rest 4 patients were induced by acute intestinal obstruction. All of these patients met the diagnostic criteria for ARDS secondary to sepsis (SAPS II score > 40; acute lung injury score > 2.5). Clinical features for the selected patients at the exudative and recovery phase are summarized in Table 1.

**Table 1:** Clinical features for the selected ALI/ARDS patients

**Description**	**Exudative phase**	**Recovery phase**
Age	48 ± 6.3	48 ± 6.3
Male/Female	10/4	10/4
P_a_O_2_/F_i_O_2_	127 ± 36.3	208 ± 13.2
SAPSII	58 ± 7.6	33 ± 2.6
Lung injury score	2.8 ± 0.59	2.2 ± 0.17

Exudative Phase: The first day of ARDS diagnosis.

Recovery Phase: The fifth day of ARDS diagnosis.

### Characteristics of BALF

The next important question is whether patients after day 5 of ALI/ARDS onset were indeed at the recovery phase, as we assumed that AMs collected at this time point are associated with antiinflammatory properties. To address this issue, we examined BALF samples for the number of inflammatory cells and the content of proteins. It was noted that the number of polymononuclear leukocytes (PMNs) and neutrophils were significantly lower on day 5 of disease onset as compared with that of exudative phase (within 24 h of diagnosis) (Table 2). Similarly, AM number was 35% lower at this stage than that at the exudative phase (Table 2). On the contrary, the content of total protein in BALF was 1.5fold higher at the exudative phase as compared with that at the recovery phase (116.2 ± 21.3 mg/dl vs. 46 ± 8.8 mg/dl), and similar results were obtained for albumin (65.7 ± 13.4 mg/dl vs. 11.5 ± 2.6 mg/dl) (Table 2). Together, our data confirm that on day 5 of ALI/ARDS onset, the patients were indeed at the recovery phase, and AMs collected at this time point should manifest antiinflammatory properties.

**Table 2:** BALF characteristics collected at the exudative and recovery phase

**Description**	**Exudative phase**	**Recovery phase**
Recovered lavage fluid (mL)	63 ± 12	58 ± 13
Total protein (mg/dL)	116.2 ± 21.3	46 ± 8.8
Albumin (mg/dl)	65.7 ± 13.4	11.5 ± 2.6
Total cell number (*10^4^/L)	88.2 ± 12.2	49.6 ± 7.8
AM number (*10^4^/L)	57.3 ± 6.8	42.6 ± 3.3
PMN number (*10^4^/L)	31.8 ± 5.6	8.9 ± 2.5

Exudative Phase: The first day of ARDS diagnosis.

Recovery Phase: The fifth day of ARDS diagnosis.

### Results for 2D PAGE

**Figure 1:**
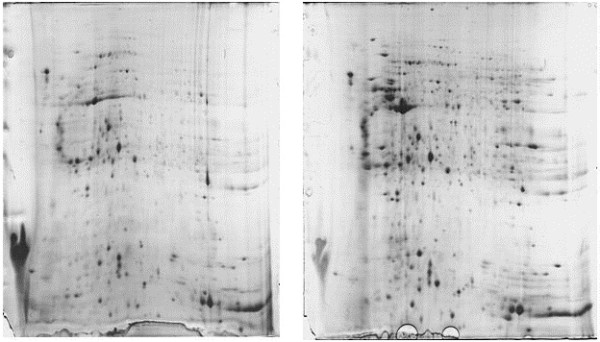
**Representative images for the 2-D PAGE results.** The studies were conducted using proteins originated from the exudative and recovery pool, respectively. Each pool was run in 3 separated gels, and an average of 1100 identifiable protein spots can be consistently characterized in each replicated gel.

Given that AMs collected from each individual patient at each time point were very limited (Table 2), we thus pooled AMs from all 14 patients at the exudative phase (exudative pool) and recovery phase (recovery pool), respectively. The pooled AMs were next subjected to protein extraction under the same condition, and about 1 mg of total proteins was obtained for each pool. 2D PAGE was then carried out using these two pooled samples. To demonstrate the reproducibility, each pool was run independently in 3 separated gels. We obtained similar results for protein distribution patterns, and Figure 1 shows the representative images of 3 runs.

### Analysis of protein spots with differential expressions

We next employed the Gel-Doc2000 image acquisition software and the PDQuest 2D analysis software to characterize protein spots with differential expression levels between the two pools. In average, around 1100 identifiable protein spots were consistently shown in each gel of the two pools. A digital ID was then automatically generated for each spot by the software. The relative expression levels for each protein spot were subsequently defined by standardized volume [V (%) = volume of each spot/total volume of all protein spots on the same gel]. Parallel comparative analysis was next carried out between the gels in the exudative pool and recovery pool to characterize protein spots with differential expressions. Only those spots showing consistent result in all parallel gels were selected, which allowed us to identify 135 protein spots with alterations between the exudative and the recovery pool. Among which, 87 spots showed ≥ 2.5 fold differences, while the rest 48 spots showed differences < 2.5 fold.

### Results for MALDI-TOF-MS and peptide mass fingerprint (PMF) analysis

**Figure 2:**
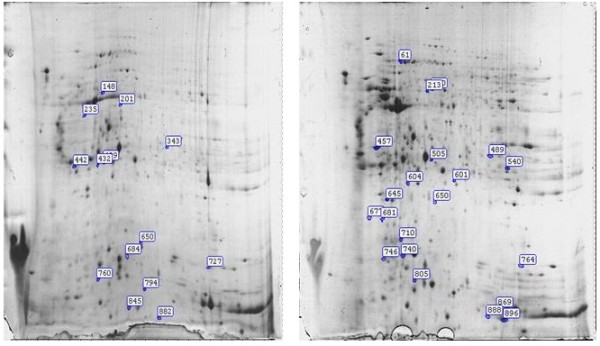
**The identity and location of 35 protein spots with good MALDI-TOF-MS results.** A total of 135 protein spots were characterized with differential expression between the exudative pool and the recovery pool, but only those 35 spots produced good quality of peptide mass.

**Figure 3:**
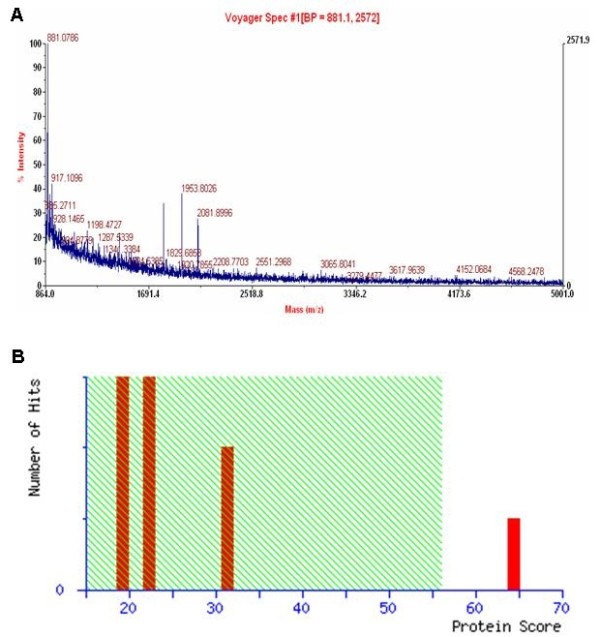
**Representative results for Peptide Mass Fingerprint (PMF) analysis. A.** MASCOT Score Histogram for protein spot 805. **B.** MASCOT database query results for protein spot 805. The spot was characterized as S100A9 by MASCOT database query (fractional value = 64, p < 0.05).

The above characterized protein spots were next excised from the gels and subjected to MALDI-TOF-MS analysis of protein identity. Unexpectedly, only 35 excised spots produced good quality of peptide mass, and their digit IDs and locations on the 2D gels are shown in Figure 2. Peptide mass fingerprint (PMF) analysis was then conducted to search databases for protein identity. Once PMF analysis characterized a match for the spectrum in the database, isotope removal was then conducted by the software MASCOT DISTILIER, and the recognized target peaks were next labeled with numbers. Figure 3A shows a typical example for MASCOT Score Histogram for protein spot 805, while Figure 3B shows the fractional value for protein spot 805 generated by MASCOT database query, and this protein ID was finally characterized as S100A9. Similar analytical process was then carried out for the rest 34 protein spots. Interestingly, we failed to characterize a reliable match in database query for 8 protein spots, and they are spots 442, 489, 681, 677, 684, 760, 845 and 888, respectively. Information for the remaining 27 spots is shown in Table 3. Specifically, spots 61, 200, 213, 457, 505, 540, 601, 604, 605, 645, 710, 740, 746, 764, 805, 869, and 896 were found to be upregulated in the recovery phase of AMs, while spots 148, 201, 235, 343, 409, 432, 650, 727, 794, and 882 showed higher levels of expression in the exudative phase of AMs.

**Table 3:** Results for proteins with differential expressions

**Spot**	**Protein ID**	**Accession #**	**Fold**	**Possible functions**
213	Actin-related protein 3c	NP-001157930	+2.8	ATP binding
457	Annexin A5	NP-001145	+3.6	Signal/inflammation
604	Gamma-actin	CAA27723	+3.5	ATP binding
645	Rho GDP-dissociation inhibitor 2	NP-001166	+4.1	Metabolism
740	Chain J	ABI63362	+4.0	Antigen binding
805	Protein S100-A9	NP-002956	+3.9	Calcium ion binding
896	Protein S100-A8	NP-002955	+4.8	Calcium ion binding
869	Peroxisome biogenesis factor13	NP_002609	+5.4	Redox
601	Peroxiredoxin-6	P30041	+2.9	Antioxidant
505	Leukocyte elastase inhibitor	NP_109591	+4.5	Related to proteolysis
540	Voltage-dependent anion-selective	P21796	+2.6	Transport channel protein 1
61	Methylenetetrahydrofolate	EAW47761	+3.1	Redox/metablism
	dehydrogenase (NADP + dependent)			
	1-like, isoform CLCP			
710	GSTP1	CAG29357	+5.8	Transferase
746	Interleukin-1 receptor antagonist	CAA37386	+3.7	Acute-phase response
	protein			
764	Tumor necrosis factor alpha-	NP_001161414	+3.2	Transcript protein8-induced protein 8-like protein1
605	6-phosphogluconolactonase	NP_036220	+2.9	Hydrolase
200	Aldehyde dehydrogenase	AAA51693	+2.7	Redox
343	Galectin-3	BAA22164	-2.9	Carbohydrate binding
409	Electron transfer flavprotein	CAB37832	-2.8	Electron carrier
	subunit beta			
882	Heat shock protein 27	AAA62175	-4.1	Inflammation/apoptosis
148	Macrophage-capping protein	AAA59570	-2.8	Actin capping
794	Napsin-A	NP_004842	-3.1	Protein proteolysis
727	Superoxide dismutase	AAB59626	-2.6	Antioxidant
235	CathepsinB	NP_680093	-3.1	Peptide binding
650	Tripeptidyl-peptidase I	O14773	-2.8	Aminopeptodrate
432	Annexin A8	AAH73755	-3.5	Calcium ion binding
201	Protein disulfide isomerase-related	AAB50217	-2.9	Redox regulation protein 5

+: Increased fold in the recovery phase; -: Increased fold in the exudative phase.

The identity and functional relevance of above 27 protein spots are also summarized in Table 3. These proteins are involved in inflammatory responses, cell skeleton organization, oxidative stress, apoptosis and metabolism. For example, cathepsin B (spot 235) was found to be highly expressed in the exudative phase of AMs, while high levels of cathepsin B has long been recognized to play a pivotal role in airway imflammatory response [14]. In sharp contrast, neutrophil elastase (NE) inhibitor or serine protease inhibitor (spot 505) was characterized to be significantly upregulated in the recovery phase of AMs, and previous studies have consistently demonstrated that NE inhibitor possesses high potency against acute lung injury [15]. Together, these data support that those proteins characterized through current study could be valuable biomarkers for assessing ALI/ARDS progression and prognosis.

**Figure 4:**
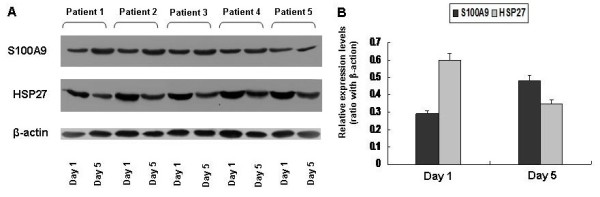
**Results for Western blot analysis of S100A9 and HSP27.** Cell lysates from AMs at the exudative phase and recovery phase originated from 5 ALI/ARDS patients were employed for the analysis, respectively. **A.** Representative results for Western blot analysis. **B.** A bar graphic figure showing the average expression levels for S100A9 and HSP27. In consistent with the 2D PAGE results, a marked increase for S100A9 was noted in the recovery phase, while HSP27 was found with much higher levels of expression at the exudative phase. β-actin was used for normalization.

To validate the above proteomic results, we randomly selected spots 805 (S100A9) and 882 (HSP27) for Western blot analysis using AM lysates from 5 ALI/ARDS patients. In consistent with the results from 2D analysis, AMs at the recovery phase consistently showed much higher levels of S100A9 expression in all patients examined as compared with that at the exudative phase (Figure 4A). In average AMs at the recovery phase manifested a 70% increase for S100A9 expressions (Figure 4B). In sharp contrast, HSP27 was found to be highly expressed at the exudative phase of AMs (Figure 4A), and in average a 75% reduction was noted for AMs at the recovery phase (Figure 4B). Together, these results provided feasible evidence for confirming the proteomic data resulted from 2D analysis.

## Discussion

In the present report, we have conducted comparative proteomic analysis to identify proteins relevant to the functionality of AMs at the exudative phase and recovery phase during the course of ALI/ARDS. Our data support that AMs undergo a functional property switch during ALI/ARDS initiation and recovery, in which AMs initiate, amply and perpetuate inflammatory responses in the early stage (exudative phase) of ALI/ARDS, while AMs manifest antiinflammatory properties to prevent further tissue injury and perpetuated inflammation in the recovery phase of ALI/ARDS. Therefore, those proteins characterized through current report could be valuable biomarkers for assessing ALI/ARDS progression and prognosis.

Of note, cathepsin B, a lysosomal cysteine proteinase, was found to be significantly upregulated in the exudative phase of AMs, which could be caused by the reduced lysosomal membrane stability, increased permeability and even membrane rupture. Given the role of cathepsin B played in enhancing inflammatory response, copious amount of cathepsin B released from AM lysosome into the cytoplasm or tissue space would promote ALI/ARDS progression by exacerbating inflammatory response in the lung, and therefore, cathepsin B could serve as a biomarker for early diagnosis of ALI/ARDS. In line with this assumption, cathepsin B has been suggested to a potential prognostic marker for inflammatory breast cancer [16].

Similarly, heat shock protein 27 (HSP27, spot 882) was characterized to be upregulated in the exudative phase of AMs, but significantly downregulated in the recovery phase of AMs. HSP27 belongs to the heat shock protein (HSP) family, which has been noted to be widely involved in many biological processes such as cell proliferation, differentiation and apoptosis [17-20]. During the course of an inflammatory response, increased synthesis of cytokines induces the expression of heat shock proteins to prevent endoreticular (ER) stress. More recently, heat shock proteins have also been recognized to be potent mediators of inflammation and immunity [21]. Particularly, similar as HMGB1, passively released heat shock proteins are considered to be innate alarmins for the initiation of tissue repair or clearance of invaded pathogens [22,23]. In the exudative phase of ALI/ARDS, activated AMs are presumed to secrets copious amount to pro-inflammatory cytokines to perpetuate inflammatory response in the lung, which requires the expression of heat shock proteins such as HSP27 to prevent ER stress. On the other hand, those induced heath shock proteins can be passively released from the damaged AMs, which then amplify or exacerbate inflammatory responses along with ALI/ARDS progression. Therefore, similar as cathepsin B, HSP27 possesses the properties to be a biomarker for ALI/ARDS early diagnosis.

Of note, unlike cathepsin B and HSP27, neutrophil elastase (NE) inhibitor or serine protease inhibitor (spot 505) was characterized to be significantly increased in the recovery phase of AMs. NE is the major protease released by PMNs during the course of ALI/ARDS. It impacts epithelial integrity to induce lung injury by digestion and degradation of the extracellular matrix. As a result, NE has been considered to be a main effector in ALI/ARDS inflammatory cascade [24]. Indeed, studies in ALI/ARDS patients have consistently revealed that NE expression is associated with disease progression. In sharp contrast, NE inhibitor attenuates the release of inflammatory mediators to effectively suppress inflammatory cytokine cascades. Particularly, it antagonizes microbial activities through inhibition of TNF-a/IL-1β expression and attenuation of NF-kB activation. Indeed, recombinant human NE inhibitor has been found to provide protection for rats against cystic fibrosis induced lung injury [25]. In line with this notion, a variety of NE inhibitors have been employed in the settings against severe infections [26]. Thus far, three categories of protease inhibitors are found to be naturally distributed in the bronchus and lung tissues, the a1-proteinase inhibitor (a1-Pi), the secretory leukocyte protease inhibitor (SLPI) and the specific protease inhibitors (Elafin). All together, it is plausible to assume that NE inhibitor could be an ideal biomarker for ALI/ARDS prognosis.

Interestingly, S100-A8 (spot 896) and S100-A9 (spot 805) were found to be highly expressed in the recovery phase of AMs as well. S100 is a group of low-molecular-weight calcium-binding proteins. Although a great deal of effort has been devoted to the studies of S100 proteins, their functional relevance is still remained obscure [27]. Torre and colleagues found that S100 proteins interact with several key factors implicated in ALI/ARDS pathogenesis including TNF-а, IL-6 and p38MAPK, but has no obvious connections with other non-inflammatory proteins [28], suggesting that S100 proteins (mainly S100A8 and S100A9) could be a potential biomarker for ALI/ARDS prognosis and a therapeutic target in the settings of patients with ALI/ARDS.

Of note, although the above identified proteins have great potential to serve as biomarkers for ALI/ARDS early diagnosis and prognosis, a simulation dataset with new ALI/ARDS patients would be essential for validation of their feasibility. Furthermore, this report only characterized 27 informative proteins, and it is likely that more proteins should be implicated in this functional switch for AMs during the course ALI/ARDS. Therefore, additional studies with more patients and advanced technologies would be necessary to further address this issue. Also, we only selectively conducted Western blot analysis for S100A9 and HSP27, and follow up studies aimed at confirming the expression changes for the rest proteins are needed.

## Conclusions

We have conducted comparative analysis of AM proteome at the exudative phase and recovery phase during the course of ALI/ARDS. Peptide mass fingerprint (PMF) analysis characterized 27 informative proteins, and 17 of which were found to be upregulated in the recovery phase, while the rest 10 were identified with higher expression levels at the exudative phase. Given that these proteins play pivotal roles in the regulation of inflammatory responses, they could have the potential to serve as biomarkers for ALI/ARDS early diagnosis and prognosis.

## Abbreviations

ALI: Acute lung injury; ARDS: Acute respiratory distress syndrome; AM: Alveolar macrophage; BALF: Bronchoalveolar lavage fluid; 2D PAGE: Two-dimensional poyacrylamide gel electrophoresis; MALDI-TOF-MS: Matrix-assisted laser desorption/ionization time of flight mass spectrometry; PMN: Polymophnuclear leulocyte; NE: Neutrophil elastase; a1-Pi: a1-Proteinase inhibitor; PMF: Peptide mass fingerprinting.

## Competing interests

The authors declared that they have no competing interest.

## Authors’ contributions

HD: conducted the experiments; YL, YZ and GW collected the patient samples; SH, XH and PY helped Western blot analysis and reference formating; JL, ZZ and XX designed the experiments and wrote the draft manuscript; CYW: helped study design and edited the manuscript. All authors read and approved final manuscript.
